# Supplementary material for: Role of heat shock protein 60 in primed and naïve states of human pluripotent stem cells
Source: PLoS One. 2022 Jun 9;17(6):e0269547. doi: 10.1371/journal.pone.0269547 (PMC9182300; doi:10.1371/journal.pone.0269547)
Supplement: S1 Table — (DOCX) [file pone.0269547.s001.docx]

**S1 Table 1. Primers used for the qPCR analysis**

| Classification | Gene name | Sequence (5'→3') | Ref |
| --- | --- | --- | --- |
| Naïve-state-specific genes | DNMT3L(F) | TGAACAAGGAAGACCTGGACG | Gafni et al, 2013 [[9](#_ENREF_9)] |
|  | DNMT3L(R) | CAGTGCCTGCTCCTTATGGCT |  |
|  | XIST(F) | AGGGAGCAGTTTGCCCTACT | Gafni et al, 2013 [[9](#_ENREF_9)] |
|  | XIST(R) | CACATGCAGCGTGGTATCTT |  |
|  | STELLA (F) | GTTACTCGGCGGAGTTCGTA | Theunissen et al, 2014 [[14](#_ENREF_14)] |
|  | STELLA (R) | TGAAGTGGCTTGGTGTCTTG |  |
|  | REX1 (Zfp42) (F) | GGAATGTGGGAAAGCGTTCGT | Theunissen et al, 2014 [[14](#_ENREF_14)] |
|  | REX1 (Zfp42) (R) | CCGTGTGGATGCGCACGT |  |
|  | ESRRB(F) | GCCATGATGGAAAATGCCCC | Duggal et al, 2015 [[8](#_ENREF_8)] |
|  | ESRRB(R) | CCAGAGATGCTTTCCCTGGC |  |
|  | PRDM14(F) | TGAGCCTTCAGGTCACAGAG | Theunissen et al, 2014 [[14](#_ENREF_14)] |
|  | PRDM14(R) | ATTTCCTATCGCCCTTGTCC |  |
|  | KLF2(F) | CACACAGGTGAGAAGCCCTA | Duggal et al, 2015 [[8](#_ENREF_8)] |
|  | KLF2(R) | GCACAGATGGCACTGGAATG |  |
|  | KLF4(F) | TACCAAGAGCTCATGCCACC | Duggal et al, 2015 [[8](#_ENREF_8)] |
|  | KLF4(R) | CGCGTAATCACAAGTGTGGG |  |
|  | KLF5(F) | CCTGGTCCAGACAAGATGTGA | Shakiba et al, 2015 [[40](#_ENREF_40)] |
|  | KLF5(R) | GAACTGGTCTACGACTGAGGC |  |
|  | TBX3(F) | CGGACATACTTGTTCCCCGA | Qin et al, 2016 [[12](#_ENREF_12)] |
|  | TBX3(R) | GCAGGGTGAGCTGTTTTCTTTT |  |
|  | DPPA5(F) | GCTCCTATTTGTACAAGCTC | Stefano et al, 2018 [[18](#_ENREF_18)] |
|  | DPPA5(R) | CAAGTTTGAGCATCCCTC |  |
| Primed-state-specific genes | OTX2(F) | CAAAGTGAGACCTGCCAAAAAGA | Shakiba et al, 2015 [[40](#_ENREF_40)] |
|  | OTX2(R) | TGGACAAGGGATCTGACAGTG |  |
|  | DNMT3B(F) | AGGGAAGACTCGATCCTCGTC | Gafni et al, 2013 [[9](#_ENREF_9)] |
|  | DNMT3B(R) | GTGTGTAGCTTAGCAGACTGG |  |
