# Supplementary figures and images for: Role of heat shock protein 60 in primed and naïve states of human pluripotent stem cells
Source: PLoS One. 2022 Jun 9;17(6):e0269547. doi: 10.1371/journal.pone.0269547 (PMC9182300; doi:10.1371/journal.pone.0269547)

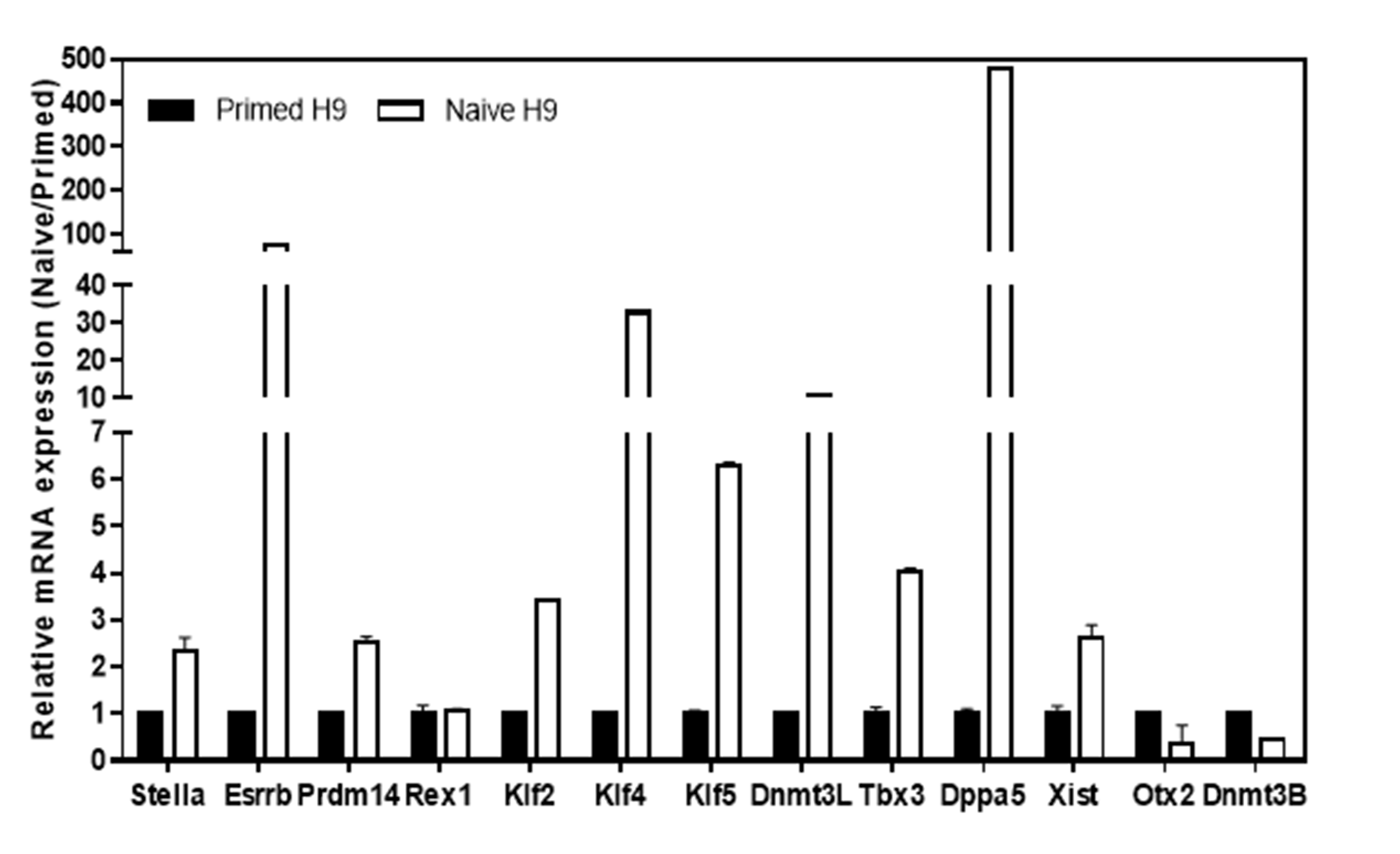

Supplement: S1 Fig — Representative primed- (OTX2 and DNMT3B) and naïve-state-specific genes (STELLA, ESRRB, PRDM14, REX1, KLF2/4/5, DNMT3L, TBX3, DPPA5 and XIST) were selected, and their expression was analyzed by qPCR. Shown is the relative expression of selected genes in 2i/L/F/A-naïve hPSCs compared to primed hPSCs. The graph represents the mean values of two independent determinations ±SD. (TIF) [file pone.0269547.s002.tif]

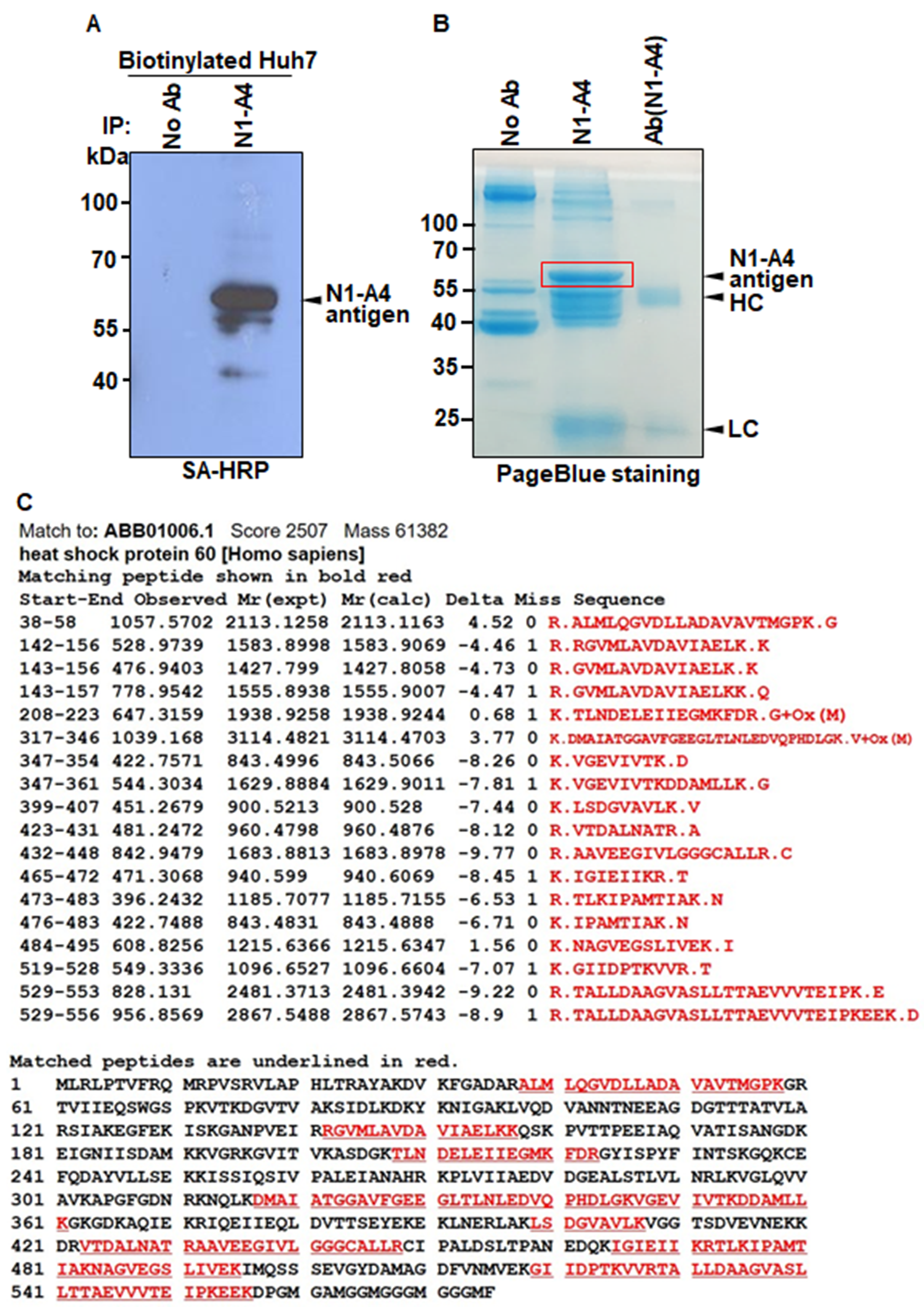

Supplement: S2 Fig — (A) Cell surface proteins of Huh7 cells were biotinylated, and the cell lysates were subjected to immunoprecipitation with N1-A4. Immunoprecipitates were detected with streptavidin-horse radish peroxidase (SA-HRP). (B) Immunoprecipitates were stained with PageBlue. The 60 kDa protein immunoprecipitated by N1-A4 is indicated by red square. (C) Mass spectrometry analysis of the 60 kDa immunoprecipitate. The 60 kDa protein band was excised and analyzed by LC-MS/MS. Eighteen tryptic peptides (bold red) originating from the 60 kDa protein matched HSP60. (TIF) [file pone.0269547.s003.tif]

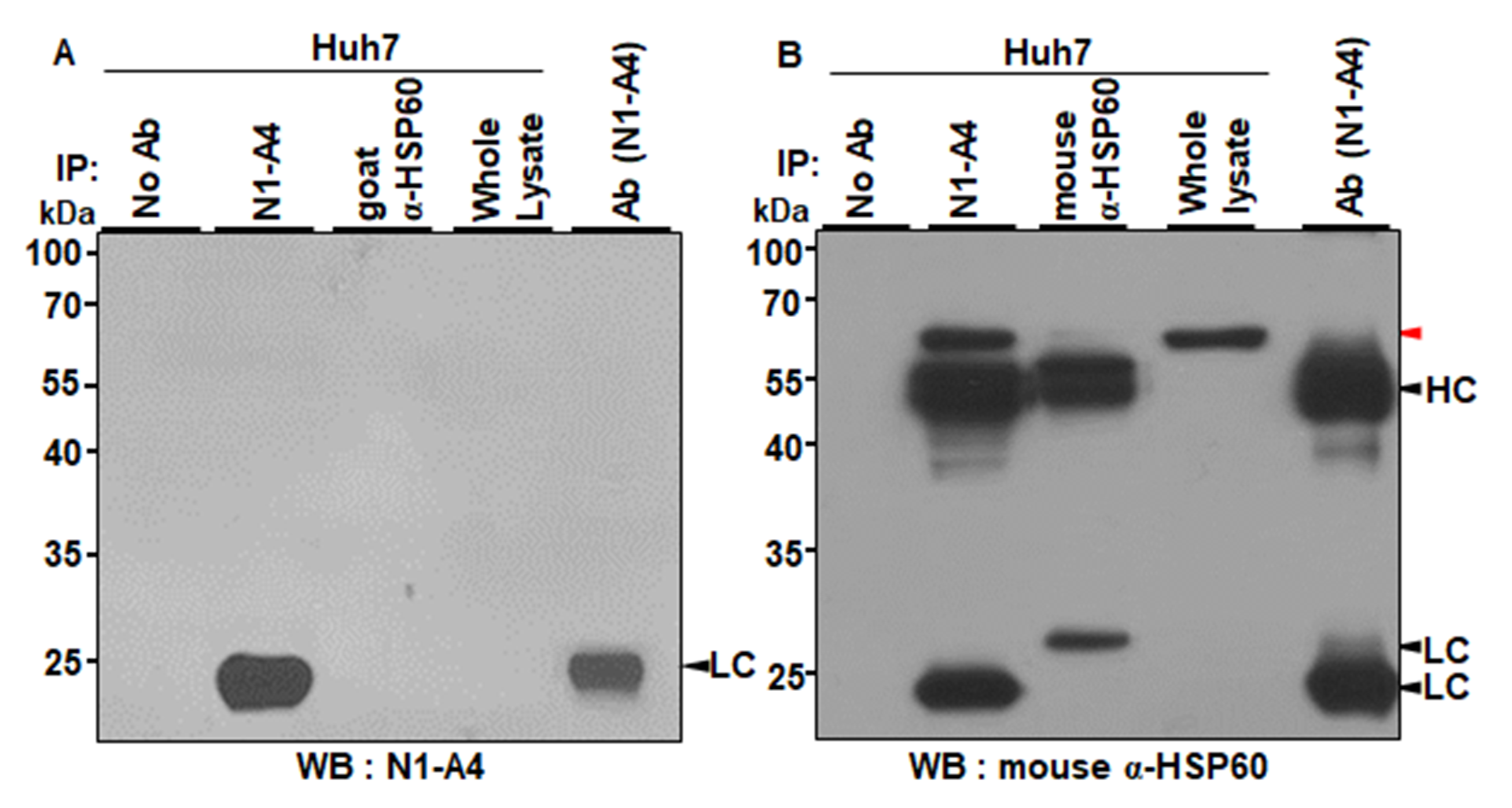

Supplement: S3 Fig — (A) Huh7 cell lysates were immunoprecipitated with N1-A4 or goat α-HSP60, and the immunoprecipitates were detected with N1-A4 in Western blot analysis. LC, immunoglobulin light chain. (A) Huh7 cell lysates were immunoprecipitated with N1-A4 or mouse anti-HSP60 antibodies (α-HSP60), and the immunoprecipitates were detected with mouse α-HSP60 in Western blot analysis. Red arrowhead indicates HSP60. HC, immunoglobulin heavy chain; LC, immunoglobulin light chain. (TIF) [file pone.0269547.s004.tif]

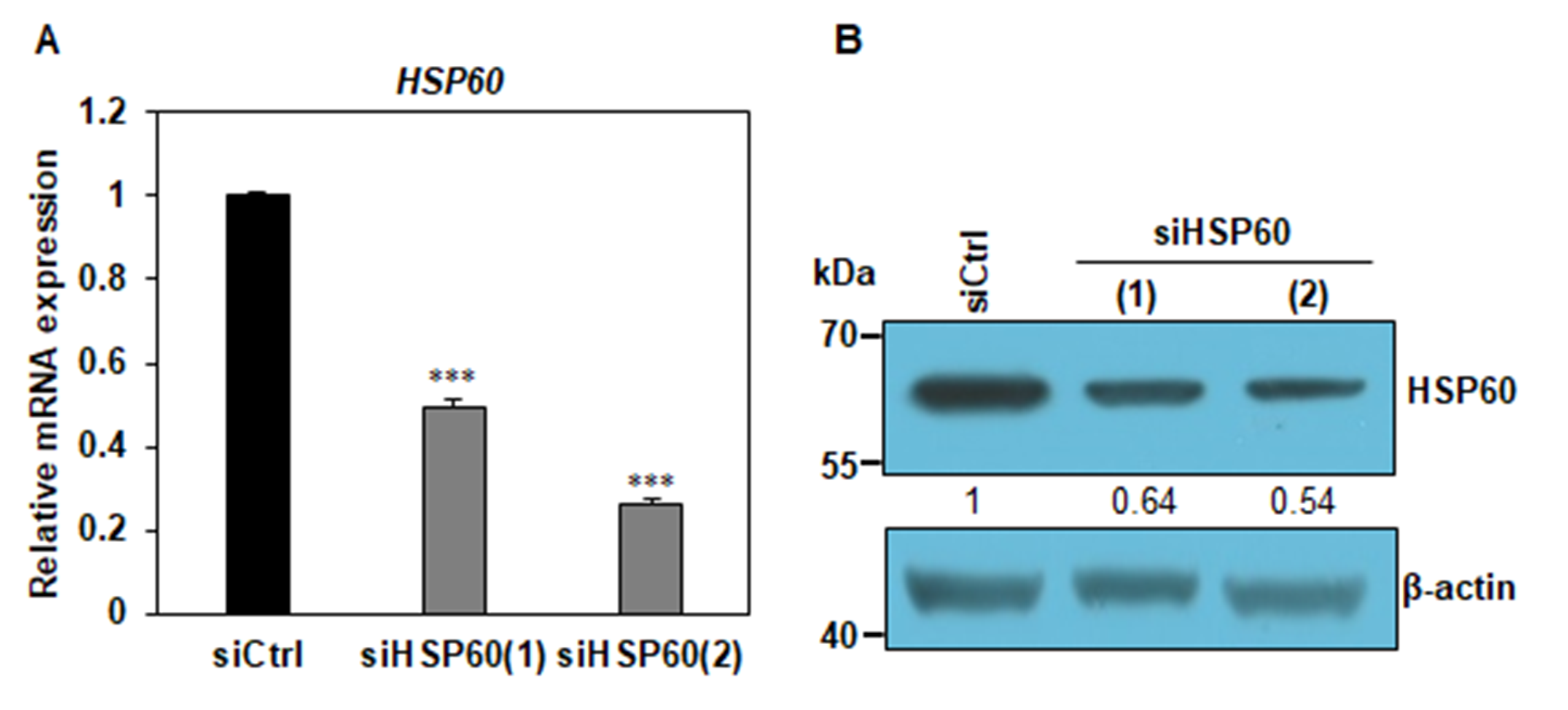

Supplement: S4 Fig — (A) Two HSP60 siRNAs were tested for HSP60 knockdown in primed H9 hPSCs. Relative mRNA levels of HSP60 were measured by qPCR and were shown after normalization against GAPDH mRNA. The graphs represent the mean values of four independent determinations ± SD (***, p < .005). (B) HSP60 proteins were analyzed in HSP60 knockdown hPSCs by Western blot analysis. Relative protein levels of HSP60 were measured using ImageJ software and normalized to the β-actin. (TIF) [file pone.0269547.s005.tif]

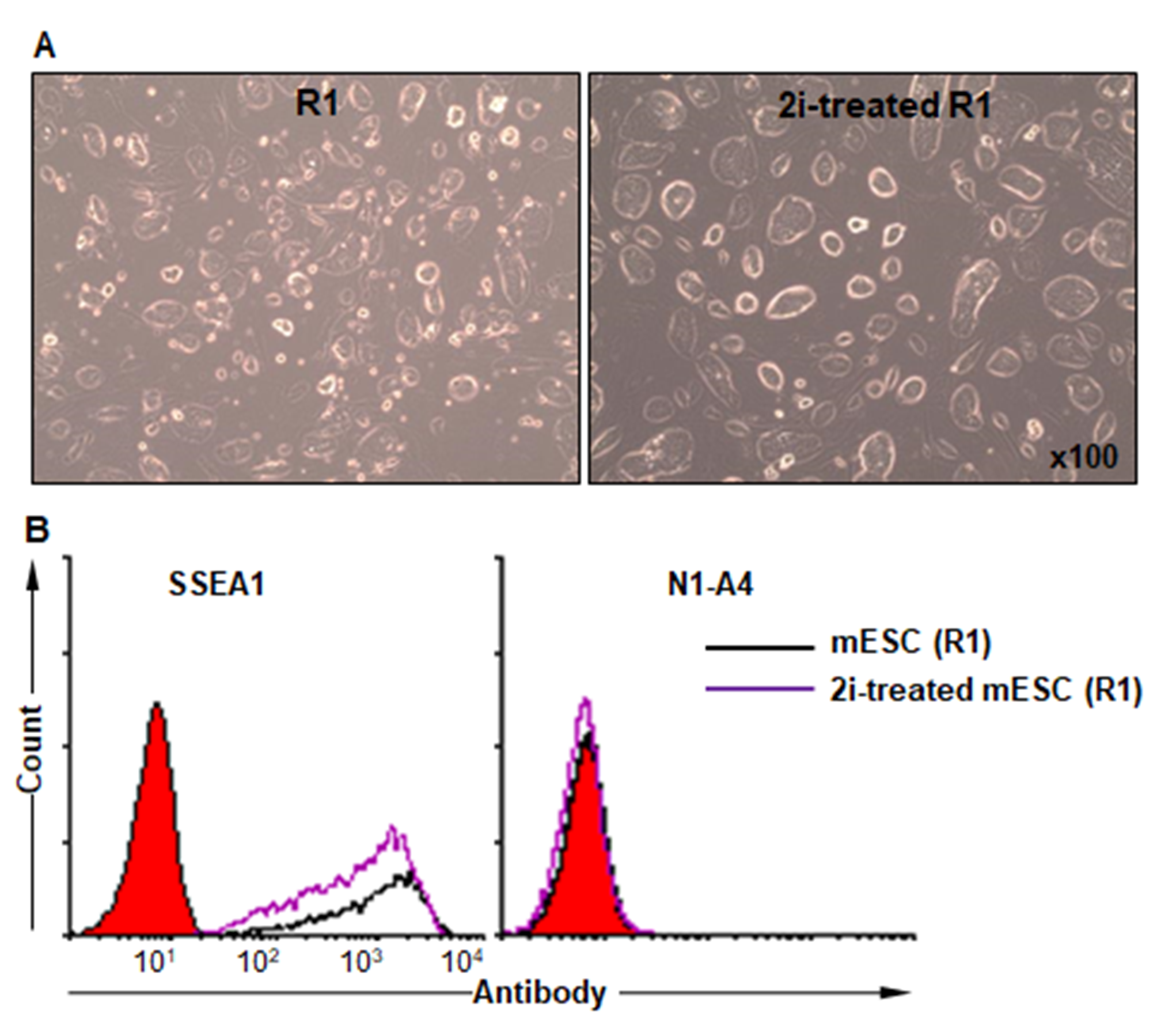

Supplement: S5 Fig — (A) Cell morphology of mESC R1 and 2i-treated R1 cells. (B) Flow cytometry analysis of SSEA-1 and N1-A4 in mESC R1 and 2i-treated R1 cells. Purple and black lines are the binding activity of the indicated primary antibody in 2i-treated or mESC R1 cells respectively. Red area represents no primary antibody control. (TIF) [file pone.0269547.s006.tif]
