# Supplementary material for: Role of heat shock protein 60 in primed and naïve states of human pluripotent stem cells
Source: PLoS One. 2022 Jun 9;17(6):e0269547. doi: 10.1371/journal.pone.0269547 (PMC9182300; doi:10.1371/journal.pone.0269547)
Supplement: S1 Raw images — (PDF) [file pone.0269547.s007.pdf]

Unprocessed scans of Western Blot and SDS-PAGE Gel of S2 Fig

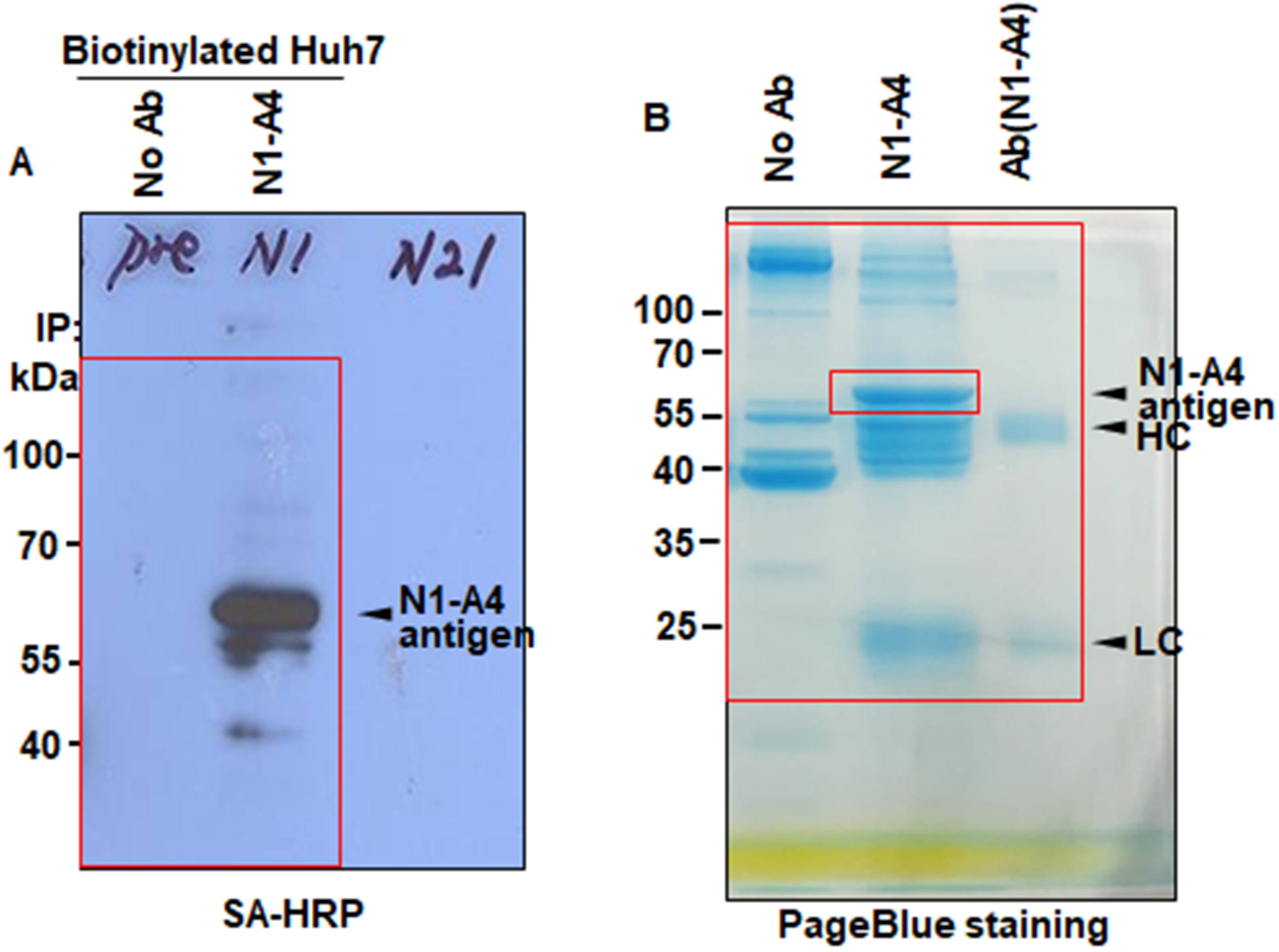

S2 Fig. Antigen identification of N1-A4.

(A) Cell surface proteins of Huh7 cells were biotinylated, and the cell lysates were subjected to immunoprecipitation with N1-A4. Immunoprecipitates were detected with streptavidin-horse radish peroxidase (SA-HRP). (B) Immunoprecipitates were stained with PageBlue. The 60 kDa protein immunoprecipitated by N1-A4 is indicated by red square..

Unprocessed scans of Western Blots of Fig 3A, 3B, 3C, and 3D

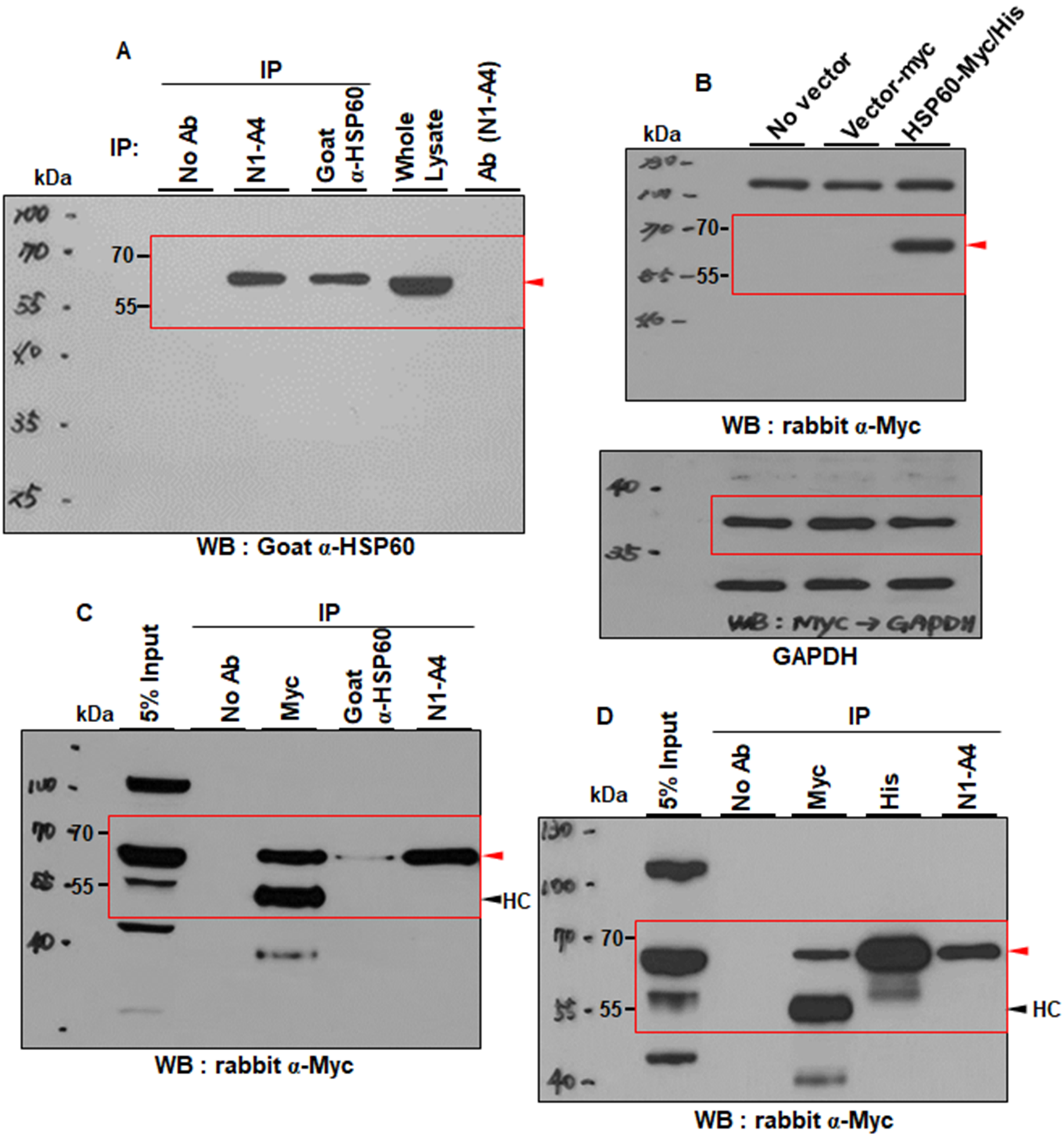

Fig 3. N1-A4 recognizes HSP60. (A) Huh7 cell lysates were immunoprecipitated with N1-A4 or goat anti-HSP60 antibodies (α-HSP60), and the immunoprecipitates were detected with α-HSP60 in Western blot analysis. Red arrowhead indicates HSP60. (B) HSP60-Myc/His vector was overexpressed in 293FT cells, and Myc-tagged HSP60 protein was detected with α-Myc. β-actin expression was the loading control. (C, D) : HEK293FT cells were transfected with HSP60-Myc/His expression vector. Cell lysates were immunoprecipitated with α-Myc, α-HSP60, and N1-A4 (C), or α-Myc, α-His, and N1-A4 (D). The immunoprecipitates were detected by Western blot analysis with α-Myc. Red arrowhead indicates the HSP60 proteins. HC, immunoglobulin heavy chain.

Unprocessed scans of Western Blots S3 Fig.

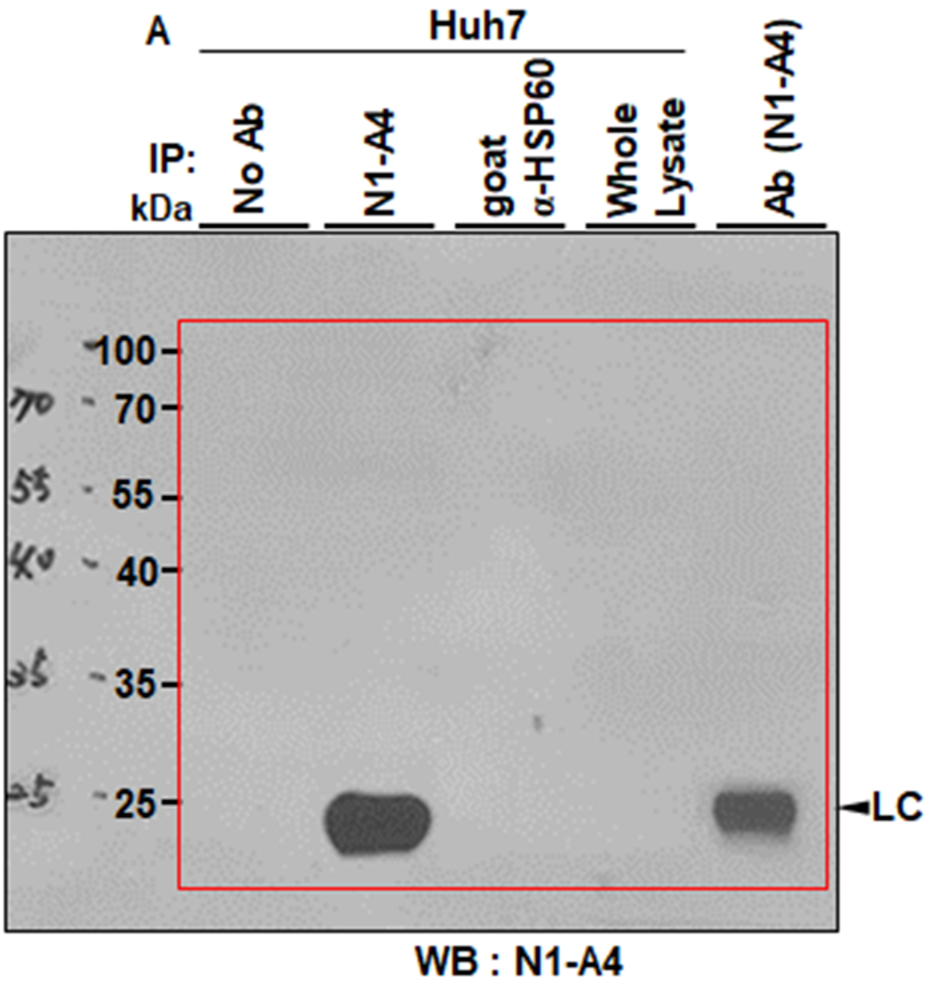

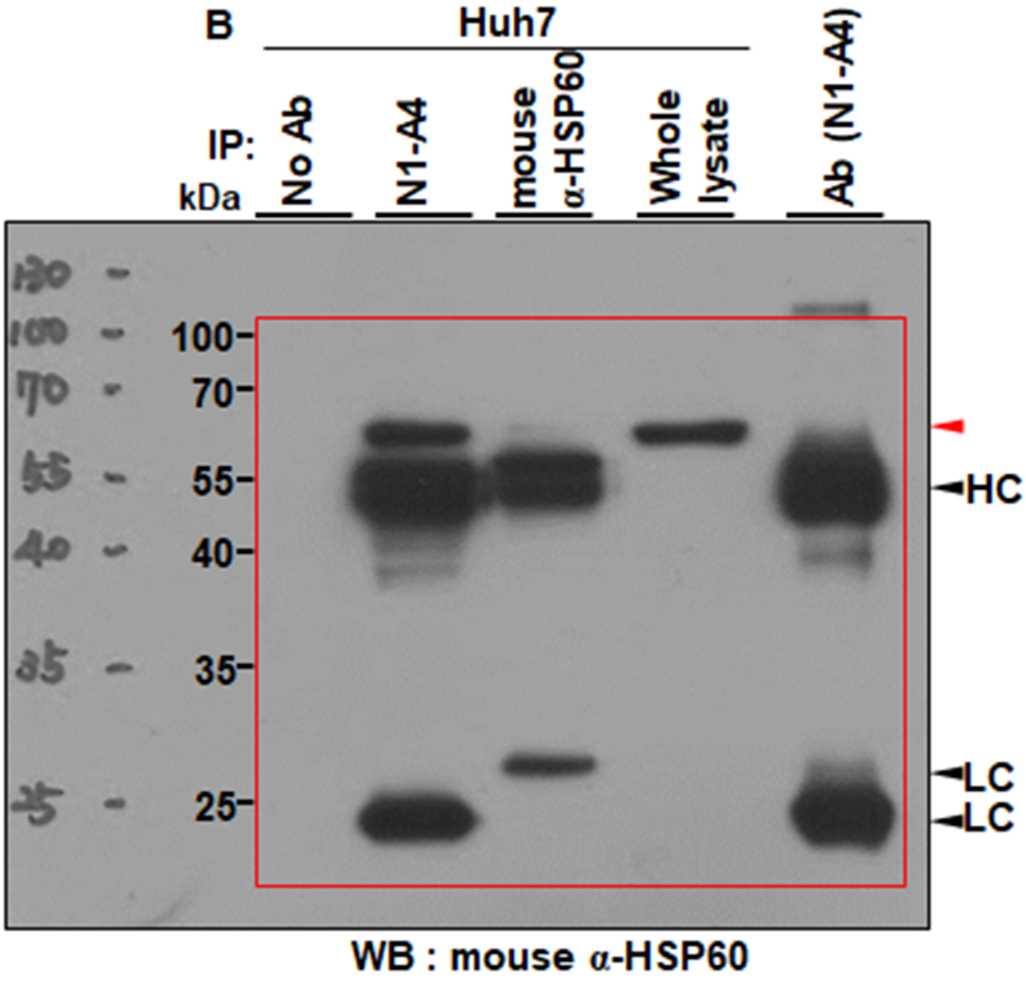

S3 Fig. N1-A4 recognizes HSP60 in a conformational dependent manner. (A) Huh7 cell lysates were immunoprecipitated with N1-A4 or mouse anti-HSP60 antibodies (α-HSP60), and the immunoprecipitates were detected with mouse α-HSP60 in Western blot analysis. Red arrowhead indicates HSP60. HC, immunoglobulin heavy chain; LC, immunoglobulin light chain. (B) Huh7 cell lysates were immunoprecipitated with N1-A4 or goat α-HSP60, and the immunoprecipitates were detected with N1-A4 in Western blot analysis.

Unprocessed scans of Western Blots of S4 Fig.

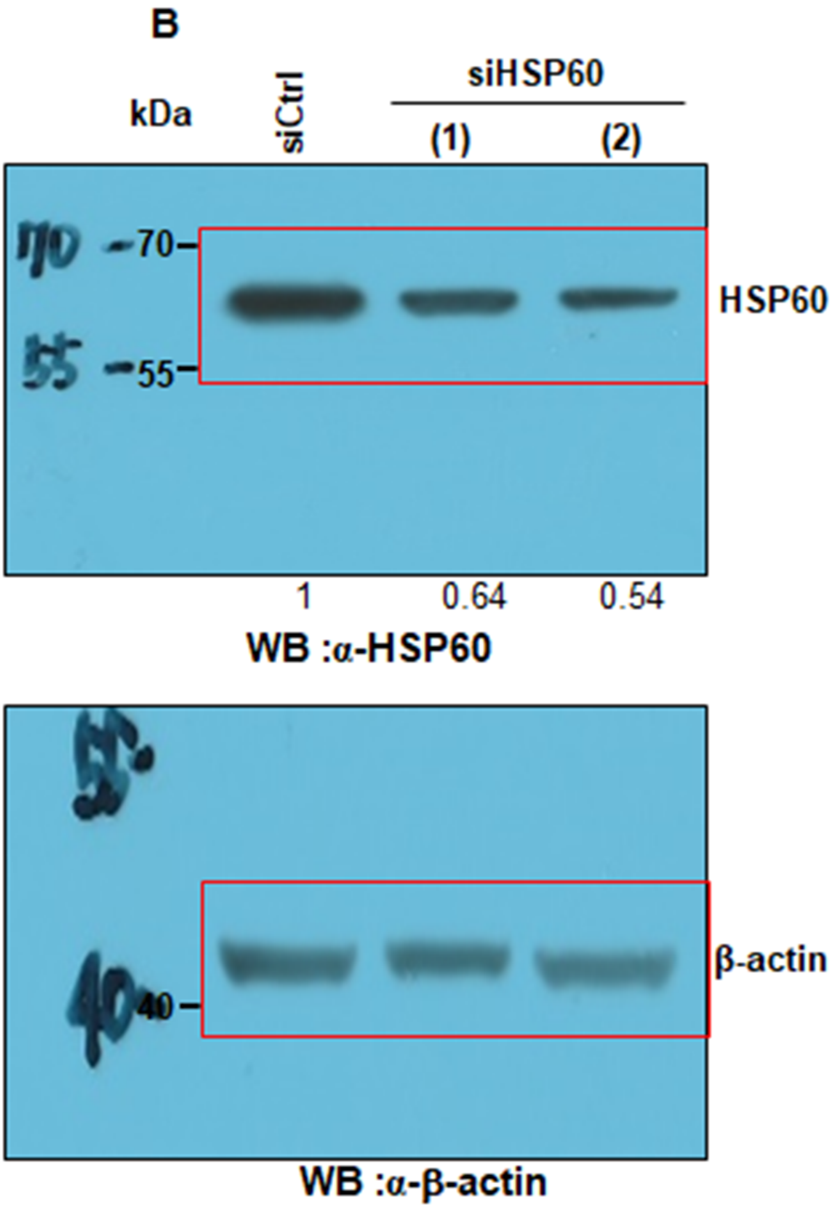

S4 Fig. Knockdown efficiency of two HSP60 siRNAs in primed H9 hPSCs. (B) HSP60 proteins were analyzed in HSP60 knockdown hPSCs by Western blot analysis. Relative protein levels of HSP60 were measured using ImageJ software and normalized to the β-actin.

Unprocessed scans of Western Blots of Fig 5A.

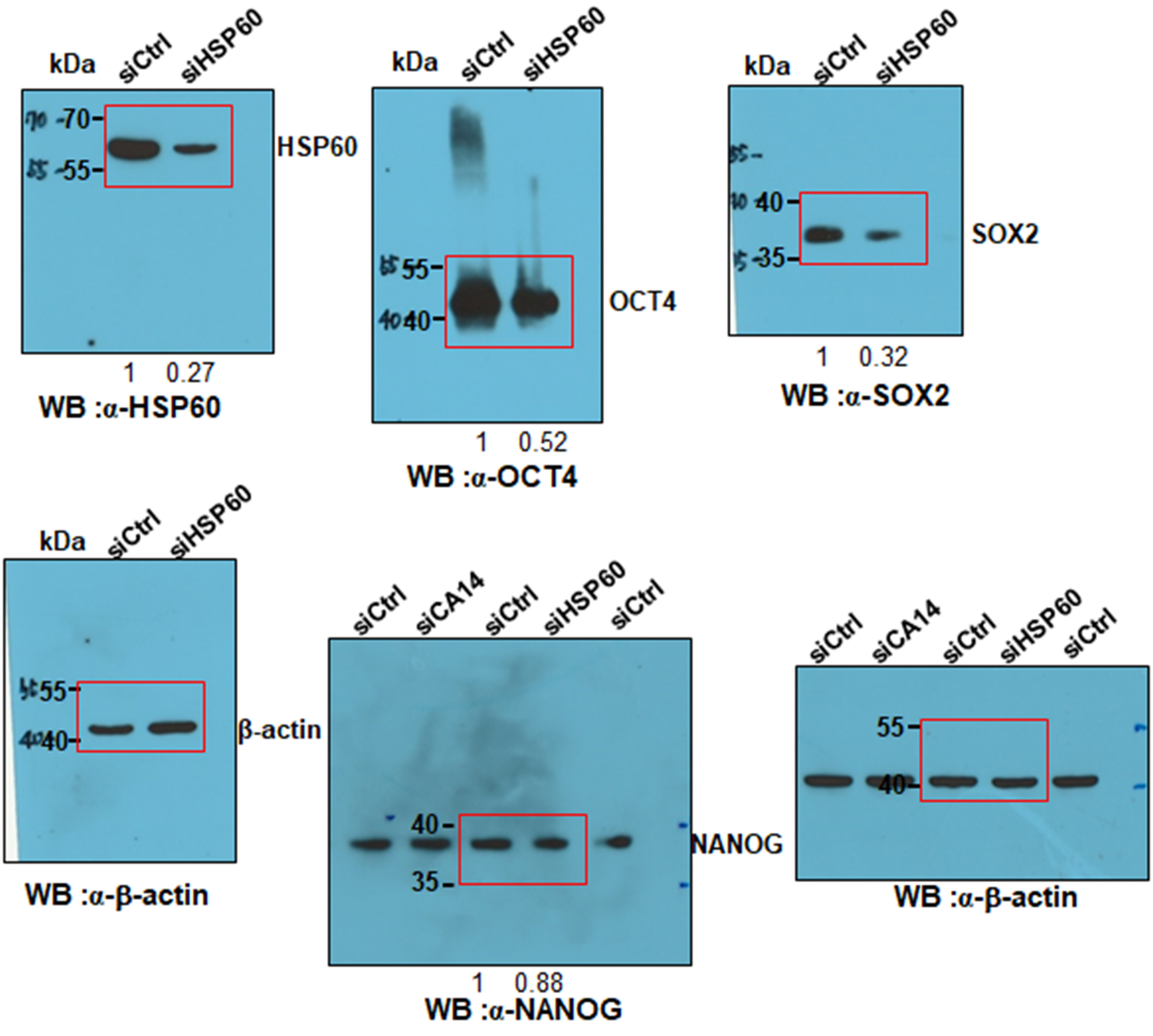

Fig 5. Knockdown effects of HSP60 in primed hPSCs. (A) Cell lysates from HSP60 siRNA-transfected H9 hPSCs were subjected to Western blot analysis with antibodies against HSP60, OCT4, SOX2, and NANOG. Relative protein levels were measured using ImageJ software and normalized to the β-actin. Shown are representative Western blots from three independent experiments.
