# Supplementary material for: Role of heat shock protein 60 in primed and naïve states of human pluripotent stem cells
Source: PLoS One. 2022 Jun 9;17(6):e0269547. doi: 10.1371/journal.pone.0269547 (PMC9182300; doi:10.1371/journal.pone.0269547)
Supplement: Attachment — Submitted filename: Reponse to reviewers.docx [file pone.0269547.s008.docx]

**Point-to-point replies**

Journal Requirements:

Please review your reference list to ensure that it is complete and correct. If you have cited papers that have been retracted, please include the rationale for doing so in the manuscript text, or remove these references and replace them with relevant current references. Any changes to the reference list should be mentioned in the rebuttal letter that accompanies your revised manuscript. If you need to cite a retracted article, indicate the article’s retracted status in the References list and also include a citation and full reference for the retraction notice.

*Response)* We checked whether all references are appropriate or not

Reviewers' comments:

Reviewer #1: The manuscript number PONE-D-22-07296

The authors carryout excellent work about importance of HSP60 in pluripotency primed and naive states. This types of research work is essential for regenerative medicine. The authors need to add some more points in discussion part, about while on differentiation states what is status of HSP60 and how it is regulates the differentiation of various cells. The manuscript scientifically very sound and well written.

*Response)* We added some more points about the role of HSP60 in PSC differentiation in discussion part. The changes that we made are red-colored in the revised manuscript with track changes.
